# Cytomegalovirus in Haematological Tumours

**DOI:** 10.3389/fimmu.2021.703256

**Published:** 2021-10-18

**Authors:** Sara Alonso-Álvarez, Enrique Colado, Marco A. Moro-García, Rebeca Alonso-Arias

**Affiliations:** ^1^ Haematology and Haemotherapy Department, Hospital Universitario Central de Asturias, Oviedo, Spain; ^2^ Laboratory Medicine Department, Hospital Universitario Central de Asturias, Oviedo, Spain; ^3^ Department of Hematologic Malignancies, Health Research Institute of the Principality of Asturias (ISPA), Oviedo, Spain; ^4^ Department of Cardiac Pathology, Health Research Institute of the Principality of Asturias (ISPA), Oviedo, Spain; ^5^ Immunology Department, Hospital Universitario Central de Asturias, Oviedo, Spain

**Keywords:** CMV, inflammation, lymphoma, transplantation, immunotherapy, CAR-T-cells

## Abstract

The exquisite coupling between herpesvirus and human beings is the result of millions of years of relationship, coexistence, adaptation, and divergence. It is probably based on the ability to generate a latency that keeps viral activity at a very low level, thereby apparently minimising harm to its host. However, this evolutionary success disappears in immunosuppressed patients, especially in haematological patients. The relevance of infection and reactivation in haematological patients has been a matter of interest, although one fundamentally focused on reactivation in the post-allogeneic stem cell transplant (SCT) patient cohort. Newer transplant modalities have been progressively introduced in clinical settings, with successively more drugs being used to manipulate graft composition and functionality. In addition, new antiviral drugs are available to treat CMV infection. We review the immunological architecture that is key to a favourable outcome in this subset of patients. Less is known about the effects of herpesvirus in terms of mortality or disease progression in patients with other malignant haematological diseases who are treated with immuno-chemotherapy or new molecules, or in patients who receive autologous SCT. The absence of serious consequences in these groups has probably limited the motivation to deepen our knowledge of this aspect. However, the introduction of new therapeutic agents for haematological malignancies has led to a better understanding of how natural killer (NK) cells, CD4+ and CD8+ T lymphocytes, and B lymphocytes interact, and of the role of CMV infection in the context of recently introduced drugs such as Bruton tyrosine kinase (BTK) inhibitors, phosphoinosytol-3-kinase inhibitors, anti-BCL2 drugs, and even CAR-T cells. We analyse the immunological basis and recommendations regarding these scenarios.

## Introduction

Human cytomegalovirus (CMV) is a DNA virus belonging to the herpesvirus family. Its transmission, through saliva, sexual contact, blood and breast milk, makes it highly prevalent, and the seroprevalence increases with age (1). The various studies carried out so far estimate a seroprevalence between 30% and more than 90%, depending on the population under study. This variation may be largely ascribed to age and socio-economic characteristics.

The implications of acute infection are of little relevance in terms of severity of infection and complications, as described below. However, the interest in healthy populations lies in its chronification and latency, and thereby in the development of an immune response that accompanies the host throughout its life, modulating its immune system through mechanisms that are not yet fully understood.

CMV infection plays a very important role in some population groups, such as immunosuppressed patients, and especially haematological patients, since acute infection causes significant morbidity and mortality in such patients.

The objective is to review the knowledge of CMV infection, and to understand its immunological effects in healthy individuals, in general, and in haematological patients.

## CMV Infection

### Primoinfection and Latent Phase

**Figure 1:**
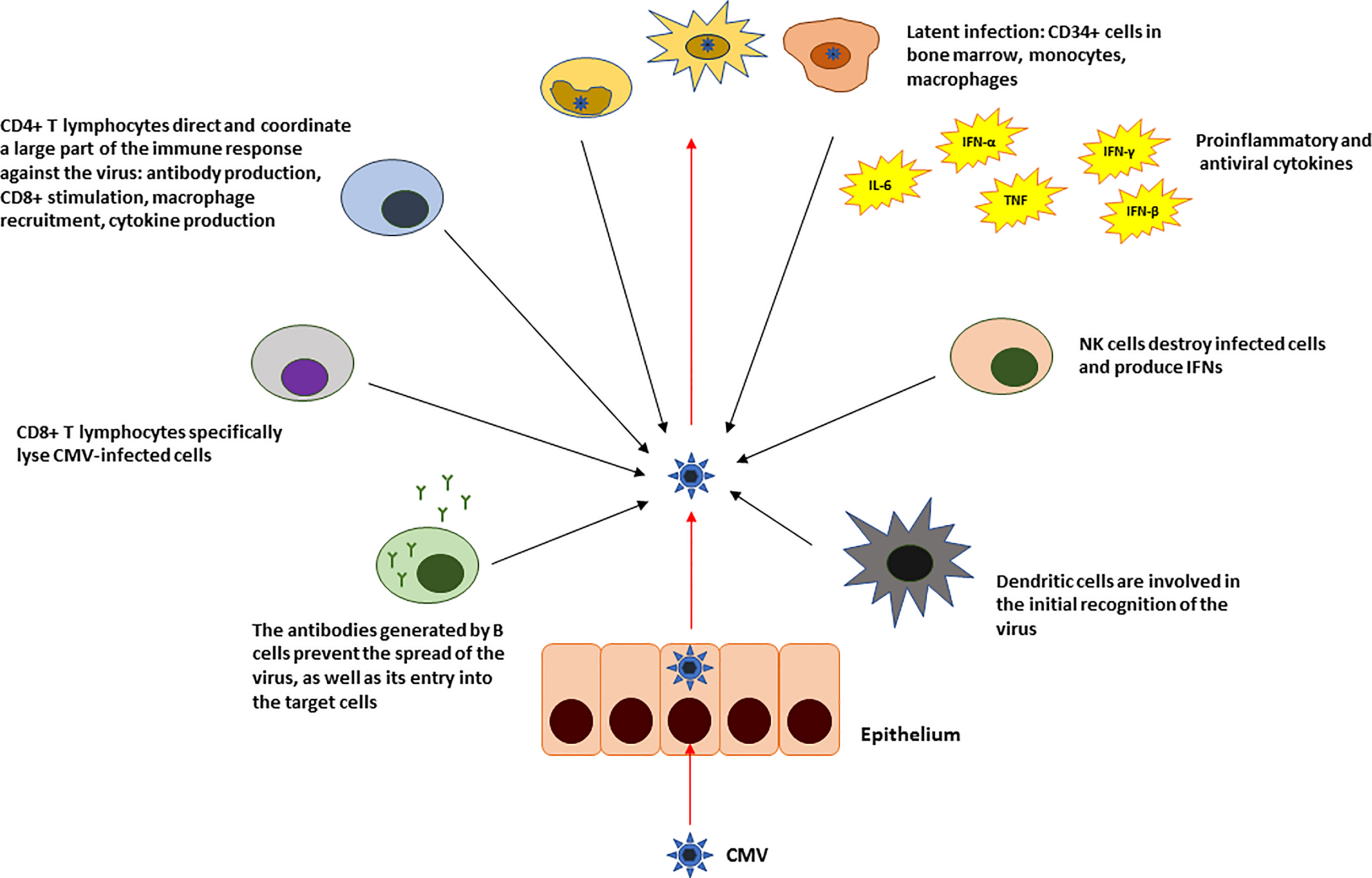
Summary of the innate and adaptive immune response against CMV in immunocompetent individuals.

The infection is usually asymptomatic in adults, although sometimes it occurs in the setting of a mononucleosis-like syndrome. CMV is able to induce a persistent infection throughout the host lifetime. This is due to its ability to remain latent in some cells. It appears that CD34+ cells and CD14+ monocytes and macrophages as well as dendritic cells may constitute the fundamental reservoir (2–4). However, viral DNA has also been found in other cells of the immune system, as well as in epithelial and endothelial cells (3, 5–7) as they get also infected and which would also explain the mechanisms by which it is transmitted (**Figure 1**).

In the infectious phase (and endogenous reactivation), the initial participation of proteins encoded by immediate-early (IE) genes is key, since they strongly activate the expression of the genes that consolidate the infectious stage (7, 8). These IE genes are expressed in cells that allow it (permissive), which are, apparently, differentiated cells (9). This expression is subject to the activation of the major immediate-early promoter (MIEP) protein. There are a variety of cellular factors that repress the activity of the MIEP in non-permissive cells (2, 10, 11). The terminal differentiation of these cells, which initially does not allow the reactivation of CMV, could decrease the expression of these factors, leaving the promoter complex active, thereby permitting viral reactivation (12, 13).

CMV infection induces specific IgA, IgG, and IgM production. Neutralizing IgGs appear early after infection and are permanently detectable thereafter. IgA can be detected for several months and even years after primoinfection (14). Cellular response is crucial in CMV infection control, CD4+ and CD8+ T lymphocytes are directed to pp65 protein and IE1 protein. When this control is well set, infection usually follows an indolent course (15).

### Chronic Infection and Possible Oncogenic Role

Long-term effects on immune system have been described despite the indolent course of CMV infection in the immunocompetent population. CMV has an impact on the T cell pool (and leaves a fingerprint) by large expansions of the CMV-specific memory pool and expansion of terminally differentiated T cells/*effector* T cells. This might impair the immunological response to neoantigens as well as the number of IL-2 and IL-4-producing CD8+ memory T lymphocytes in the elderly. CMV-specific CD8+ T lymphocytes producing IFN-γ, might contribute to a proinflammatory status, but this is probably less clear. Therefore, some aging-associated processes might be accelerated due to latent CMV infection (15, 16). However, the role of CMV on aging is, in any case, a current matter of debate. And a recent extensive review of this matter by Jackson et al. has revealed that, according to existing data, there is only limited evidence supportive of the formation and maintenance of a large population of CMV specific CD8+ T cells, known as “memory inflation”, as a mechanism of *immunosenescence* (17).

Potential oncogenicity has also been studied, yielding controversial results in solid and haematological tumours. This may be because the multiple causes, over and above many of the fundamental processes responsible for the development of solid and haematological tumours, make it difficult to assess the specific value of the individual primary causes.

The oncogenic role of other viruses and pathogens (hepatitis C and B, papilloma, and the bacterium *Helicobacter pylori*) is better established in the context of some solid neoplasms, such as the liver in the case of the first two, the cervix and larynx in the second, and stomach cancer in the case of *H. pylori*.

The association between CMV infection and numerous solid tumours in relation to its oncogenic and immunomodulatory roles has also been sought and found. In the case of colon cancer, the association between CMV and the development of cancer varies from study to study (18–21), whereby some find an association but others confirm the absence of one. A recent and very detailed review of US28 potential roles in aherosclerotic disease and cancer demonstrates the difficulty of attributing the causation of US28 in carcinogenesis and its role in atherosclerotic disease (22). In this field, the results of the investigations are now very numerous and not always concordant, so they must be integrated in order to establish consistent concepts regarding the functions of this protein. This could later relate it to CMV infection, and this molecule might then be used as a therapeutic target or disease marker and targeting US28 might prevent CMV disease and could benefit immunosuppressed individuals, including transplant patients (23).

CMV is expressed in most human glioma samples (24). However, correlation with peripheral blood CMV detection in glioblastoma patients is variable (25). The low incidence of glioblastoma cases compared with the high prevalence of CMV infection makes it difficult to explain the initiating role of CMV in the development of this neoplasm. In addition, anti-CMV treatment with antiviral drugs such as foscarnet or valganciclovir has not definitively been shown to improve survival in patients with glioblastoma multiforme (26–28). The efficacy of anti-CMV immunotherapy (29) may be due to targeting of CMV-expressing cells that drive tumour growth, activation of other immune cells that cause additional killing of CMV-negative cells, or cross-priming after killing of CMV-positive tumour cells. Therefore, a proposed role for CMV in gliomagenesis is most likely to be associated with an as yet undefined event (30), although it seems that it might be supported by CMV’s oncoimmunomodulatory role.

Although CMV is the virus whose impact seems to be the most significant with respect to T lymphocyte deregulation, it has not been given so much importance from the haematological point of view, and very little is known about its oncogenic role. In fact, very few studies have analysed the influence of CMV on haematological pathology. In contrast, the association of the Epstein–Barr virus with the development of lymphomas is well established (31–34). The fact that the EBV tropism occurs in the B lymphocytes, where it remains latent (35–37), may make the relationship much more direct for the etiological study. In the case of CMV, no indirect association has been sought, perhaps because, although not directly, the effect on, or damage caused to the functionality of B lymphocytes and, especially T lymphocytes, by chronic infection and successive reactivations indirectly affects degree of predisposition to lymphomagenesis.

Although no prospective studies have been carried out, at least three retrospective studies have analysed the influence of the virus in populations that develop lymphomas. Two of the studies did not find a higher seroprevalence in patients with T cell lymphomas compared to the age-controlled population (38, 39). However, another study (40) found a very high seroprevalence in patients with mycosis fungoides and Sézary syndrome (**Table 1**).

**Table 1:** CMV role in lymphoma development.

Study	Aim of study	Results
Gupta et al. (38)	Seroprevalence SS/MF vs Non-SS/MF	SS/MF 60.4 % (N=53) Non-SS/MF 61.5% (N=26)
Ballanger et al. (39)	Seroprevalence SS, MF& control group	Control group 37% (N=124) MF 66.67% (N=27) SS 42.86% (N=21) *p*=0.009
	PCR in affected tissue	CMV was not detected in diagnostic biopsies. CMV was detected in two SS skin biopsies realized at an advanced stage
Herne et al. (40)	Seroprevalence SS/MF vs bone marrow donors	Control group 57.3% (N=1322) MF/SS 97.4% (N=116) *p*<0.05
	Subanalysis with age-matched subgroups	CTCL 93% (N=32) Control group 53.6% (N=1103) *p*<0.05
Mehravaran et al. (41)	PCR in affected tissue IE1 (active replication)	IE1 detected in 1/25 Non-HL
	Nested-PCR in affected tissue UL138 (latency) Hodgkin and No Hodgkin	UL138 in 5/25 Non-HL and 1/25 HL

SS, Sézary Syndrome; MF, Mycosis Fungoides; CTCL, Cutaneous T-cell lymphoma; HL, Hodgkin lymphoma.

The expression of proteins and transcription factors of the virus has also been observed in some series of patients. Specifically, in a series of Iranian patients, the expression of UL138 mRNAs (as latent infection markers) and IE1 proteins (as reactivation markers) was studied by RT-PCR in patients with Hodgkin and non-Hodgkin lymphomas. The expression of UL138 mRNAs was found to be expressed in 20% of the T cell lymphomas in the series (41). Of note, this observation remains to be explained since T cells are not infected by CMV; casualty seems to be difficult to demonstrate here due to a possible indirect effect; indeed we are yet to know if a potential immune dysregulation motivated by a chronic exposure to the antigen might be responsible of this association.

## CMV in the Haematological Setting

### CMV in Allogeneic Stem Cell Transplantation

#### Definitions Regarding CMV Infection 

Definitions of CMV infection and disease were initially developed and published as part of the proceedings of the 4th International CMV Conference in Paris in 1993 and have been progressively updated, most recently in 2020 (42–45).

Infection involves the detection of CMV in biological samples. When monitoring patients after transplant, it is usually determined in blood. In these cases, it is worthwhile differentiating whether the infection is detected by finding the antigen (antigenemia), growth in cell culture (viremia), or detecting DNA (DNAemia).

Primary infection takes place in seronegative patients, while reactivation refers to virus detection in previously seropositive patients. Recurrent infection refers to the detection of CMV in a patient with evidence of infection, but when there has been a 4-week infection-free gap between the two determinations. Reinfection refers to a new infection by a different viral strain, while reactivation is established when the same viral strain, of endogenous origin is involved.

CMV disease involves the conjunction of signs and/or symptoms that indicate organ involvement (pulmonary, gastrointestinal, hepatic, retinal, renal, myocardial, encephalic, pancreatic, etc.) together with the detection of CMV (using one or more of the validated techniques) in the affected organ or tissue.

#### T Lymphocyte Reconstitution and CMV: Cause and Consequence

**Figure 2:**
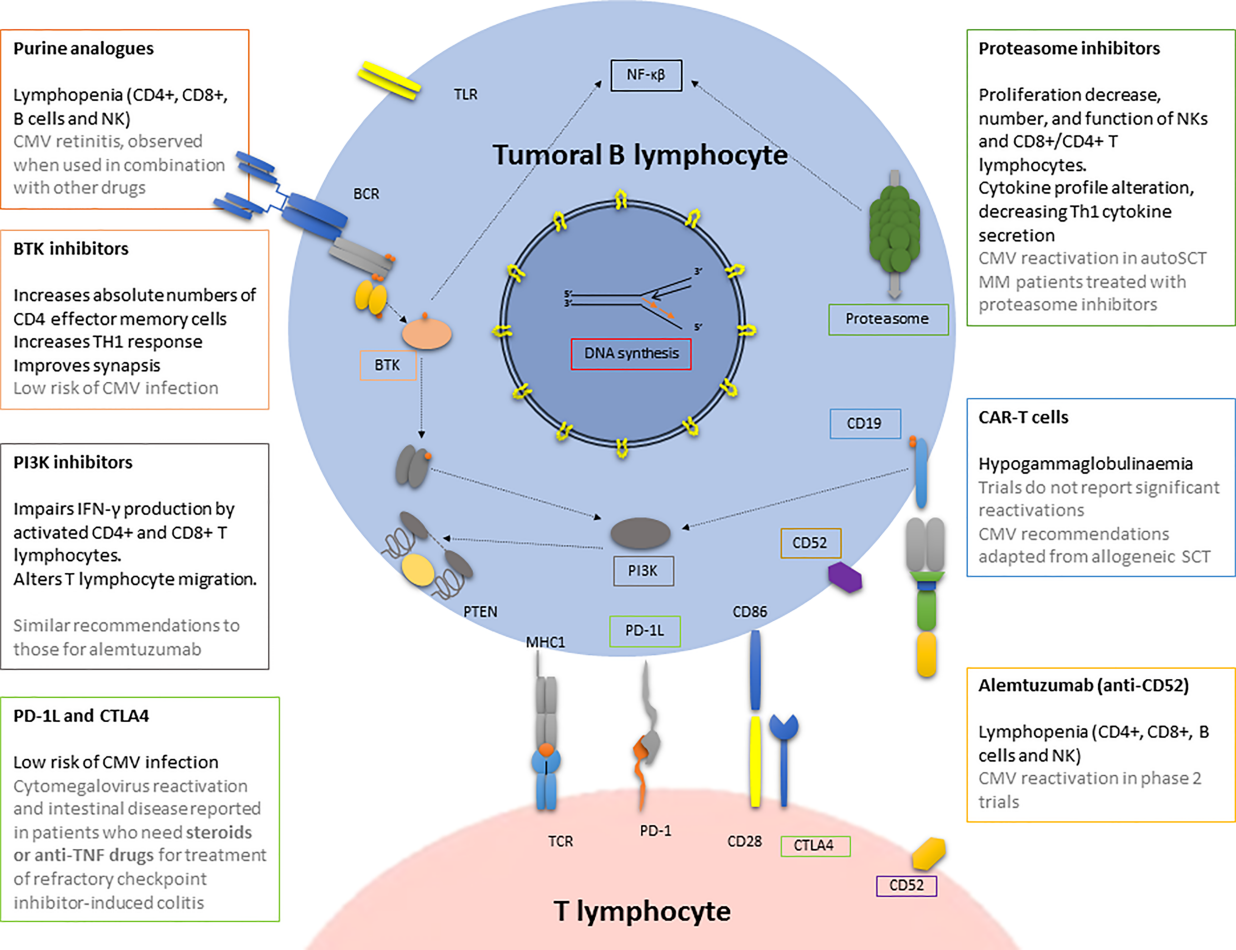
CMV infection/reactivation in the context of antitumoral drugs used in haematological patients. BCR, B cell receptor; BTK, Bruton tyrosine kinase; CAR-T cells, chimeric antigen receptor T cell; CMV, cytomegalovirus; CTLA4, cytotoxic T-lymphocyte antigen 4; MHC, major histocompatibility complex; NK, natural killer; PI3K, phosphatidyl inositol 3 kinase; PD, programmed death; PD-1L, programmed death-ligand 1; PTEN, phosphatase and tensin homologue; SCT, stem cell transplantation; TLR, toll-like receptor.

T lymphocyte reconstitution has an initial thymus-independent phase, during which we observe the antigen-driven expansion of T lymphocytes infused with the graft (**Figure 2**). The second phase is thymus-dependent. Naïve T lymphocytes derived from the donor with a diverse T lymphocyte receptor (TCR) repertoire expand, although very slowly, so it takes years to complete the reconstitutions of this subset (46). However, the process cannot always be completed in this way because thymic function is conditioned in many allogeneic SCT recipients, thymic involutes in old patients and graft-versus-host disease (GVHD) damages epithelial thymus cells (47). When this occurs, the thymic-independent pathway rapidly generates CD8+T lymphocytes (48), resulting in an inversion of the CD4:CD8 ratio that can persist for years (49). It also leads to peripheral expansion of memory T lymphocytes (CD45RO+CD27+/CD45RO+CD27-) since generation of naïve T lymphocytes (CD45RA+/CD28+) from prethymic progenitors depends on a functional thymus (50). The differences between precursor sources, conditioning regimens and donor features are a consequence of the absolute numbers of CD4+ and CD8+ T lymphocytes infused. In any case, the absolute number of CD4+ T lymphocytes (i.e., regulatory T lymphocytes (Tregs) and conventional CD4+ T lymphocytes), remain at unrecovered levels up to 2 years after hematopoietic stem-cell transplantation (HCT). CD8+ T lymphocytes can recover faster, but also depend on the conditioning regimen and immunosuppressors used. The TCR repertory is considered to drive and be the result of disease control and GVHD activity. With regard to disease control, a study including umbilical cord blood donors (UCDs) and matched related or unrelated donors using *in vivo* T cell depletion (TCD) showed that patients who remained in remission had greater TCR diversity. A broader TCR spectrum could have an antitumoral role. A narrower TCR spectrum is in turn observed in those with GVHD, which would presumably be related to preferential expansion of particular T lymphocyte clones (51). In contrast, another study reported that grade 2–3 acute GVHD is associated with greater TCR diversity (52). These differences might be due to the high variability among the conditioning regimens, immunosuppressive therapy, and donor source (53).

Activity of T lymphocytes (CD4+ and CD8+) is essential for the control of CMV infection (15, 53, 54). The correct reconstitution of CD8+ and CD4+ T lymphocytes is associated with the control of CMV infection (55). CMV-specific CD8+ T lymphocyte reconstitution is usually delayed by around 3 months after transplantation. There have been reports of prompt recovery, which is thought to be based on the receptor’s ability to bring about T lymphocyte lymphopoiesis (56). Knowing the HLA-typing of the donor, the source of the graft and the type of conditioning are essential for this rapid recovery and therefore the early control of the infection: HLA-typing facilitates the reestablishment, which is faster in identical donors without mismatch, but slower the more T depletion there is. Receptor immunity also influences infection control until immune reconstitution is established, especially in patients on reduced intensity regimens (57).

The relation between T lymphocyte reconstitution and CMV infection is bidirectional, and while delayed T lymphocyte reestablishment compromises anti-CMV protection, prompt reactivation of this virus conditions the characteristics of the recovery (58). CMV expands effector memory T lymphocytes, causing a linked contraction of all naïve T lymphocytes, including putative CD31+ thymic emigrants (59).

### Donor Source Role

With respect to the source of the graft, patients who receive an allogeneic umbilical cord blood (UCB) transplant are at the highest risk of CMV infection because of the type of cells present in UCB. In this type of transplant, T lymphocytes are immature, which lower the risk of developing GVHD, but could increase the risk of severe infections (60). UCB contains Tregs, which are more potent suppressors than adult Treg (61). On the other hand, this is consistent with the high dose of anti-thymocyte globulin employed in the conditioning regimen, which is needed to prevent mainly graft rejection but also the effects of GVHD. In summary, T lymphocyte reconstitution is delayed, and this is considered the main cause of the increased risk of infection, particularly by CMV, in this cohort (62).

Patients who receive CD34+ (positively selected) allogeneic SCT or T lymphocyte-depleted allogeneic progenitors belong to the high-risk group for CMV infection because they lack the mature and naïve T lymphocytes that should make cellular immunity reconstitution possible.

The faster engraftment of peripheral blood progenitors compared with bone marrow and cord blood progenitors, and the concordant faster immune reconstitution demonstrated *in vitro* (63) and *in vivo* (64), lead us to expect that better immune reconstitution against CMV would reduce the incidence of CMV disease in this group. Several studies have compared the sources and found mixed results for the risk of CMV reactivation in peripheral blood and bone marrow. For example, a non-randomized study of 158 patients showed that the incidence of CMV reactivation (monitoring antigenemia) and CMV interstitial pneumonia were lower in the peripheral blood than in the bone marrow group (65). Another randomized study (n = 172) showed the opposite relationship, with a higher incidence of CMV infection in unmodified peripheral blood SCT recipients (66). This latter theory of a higher risk of CMV infection in peripheral blood than in UCB and bone marrow has been described (63). However, another study found no such differences (64).

This risk of CMV infection drops to an intermediate level as time goes by, as long as anti-T drugs or high-dose steroids are not added.

The influence of the HLA discrepancy between donor and recipient is highly nuanced or dependent on the type of conditioning and immunosuppressive treatment used since these are adjusted based on these discrepancies. The greater the discrepancy, the greater the risk of viral reactivation, but as explained above, this could be a response to the confluence of a more powerful immunosuppressor therapy and the anti-T-lymphocyte agents used.

Patients undergoing haploidentical allogeneic hematopoietic SCT (Haplo-HSCT) have been considered to be at higher risk of CMV reactivation than those receiving HLA-matched allografts due to impaired CMV-specific T lymphocyte reconstitution. Analysis of this and monitoring the CMV DNA load in parallel with CMV-specific IFN-γ-producing CD8+ and CD4+ T lymphocytes revealed that CMV was reactivated approximately as often in PTCy-haplo and HLA-matched recipients, and that CMV-specific T lymphocyte counts were similar in the two groups at most of the times examined. These findings suggest that the two groups reconstitute CMV-specific T lymphocyte immunity in a similar fashion (67).

### Immunosuppressors and Anti-T Lymphocyte Agents

Steroids and anti-T lymphocyte agents are the cornerstone of the prevention and treatment of graft rejection and, fundamentally, of the development of graft-versus-recipient disease, the appearance of which considerably increases transplant-related mortality (not associated with relapse) (TRM). These data are well established, and by way of example, in one of the most recent series of patients treated with novel immunosuppressive treatment options, an increase in mortality of 5 to 16% has been described in patients with or without GVHD (68).

Patients who receive an allogeneic transplant from any source and who are treated with high-dose steroids or who receive anti-T lymphocyte agents, such as the aforementioned anti-thymocyte globulin, but also high doses of cyclophosphamide are also at high risk. This drug is necessarily employed in haploidentical transplant after progenitor infusion to avoid the GVHD that would accompany a half-discrepant haplotype. However, T lymphocyte *in vivo* depletion with cyclofosfamide is being used increasingly often in patients at high risk of GVHD when they undergo non-haploidentical transplants.

#### Cyclophosphamide 

The effects of cyclophosphamide as a T lymphocyte depletion regimen were first studied in the haploidentical transplant setting (69). Its benefits were then extended to other mismatched, or even matched, donors (70). Cyclophosphamide is used as an agent that depletes donor T lymphocytes *in vivo*. For this purpose, it is administered during the first days of infusing the progenitor cells – the post-transplant-cyclophosphamide (PTCy) strategy. The mechanism by which cyclophosphamide modifies the T lymphocyte response and thereby reduces GVHD has been studied and extrapolated based on murine models of skin graft rejections. However, there has been some disagreement about whether the mechanisms involved are similar.

Nunes et al. developed a murine major histocompatibility complex (MHC)-haploidentical HCT model (B6C3F1 → B6D2F1) that is equivalent to the clinical HCT setting. They described how PTCy, a non-T lymphocyte-cycle-dependent alkylator, affects both highly and lowly proliferative host-alloreactive donor T lymphocytes. After infusion of progenitors, host-alloreactive donor T lymphocytes become activated, proliferative, and give rise to an inflammatory environment. Between post-transplant days +3 and +7, there is continued high-level proliferation of host-alloreactive donor CD8+ effector T lymphocytes and reduced, but continued proliferation of the surviving host-alloreactive donor CD4+ T lymphocytes, both effector and Tregs (70). This time schedule and pattern of proliferation is important for determining when to apply PTCy, because the decrease in host-alloreactive donor CD4+ effector T lymphocyte proliferation is needed to prevent GVHD (71). Around day +5, the functionality of surviving host-alloreactive donor effector T lymphocytes becomes impaired. The severity of this increases over time, and the apparently rapid effect of PTCy is enhanced by preferential reconstitution of donor CD4+ Tregs between days +7 and +21, which suppresses the host-alloreactive donor effector T lymphocytes. Meanwhile, host-non-alloreactive donor T lymphocytes maintain the slow proliferation, so the relative proportion of alloreactive donor T lymphocytes ends up increasing. The dynamics after these first stages might change over time and due to antigenic stimulation.

It has been observed that the regulatory lymphocytes of patients who receive PTCy recover quickly during the post-transplant period; as little as 1 month after the transplant, they are already at levels similar to the baseline of the donor, even when the transplant patient still exhibits lymphopenia (72). In patients receiving PTCy as the sole prophylaxis of GVHD in identical transplants, it has been observed that recipients’ TCR level after infusion of the progenitors is lower than that of the donor in the first moments. However, beyond the first 3 months, it begins to resemble the donor’s repertoire more closely, and in CMV-positive cases, the number and repertoire increasingly resemble those of the donor (73).

PTCy continues to prove to be one of the most beneficial agents for the control of GVHD and even of relapse. In a prospective multi-centre, randomized phase II clinical trial, regimens of (i) tacrolimus, mycophenolate mofetil, and cyclophosphamide, (ii) tacrolimus, methotrexate, and bortezomib, and (iii) tacrolimus, methotrexate, and maraviroc were compared against standard tacrolimus and methotrexate (74). Only the PTCy-containing regimen resulted in superior GVHD-free (severe acute and chronic), relapse-free survival. However, this benefit might alter when CMV infection appears. As previously stated, an increased CMV infection is associated with Haplo-HSCT receiving PTCy (HaploCy). However, the specific roles of the allograft source and the use of PTCy in CMV infection and disease are unresolved. A recent analysis of patients reported to the Center for International Blood and Marrow Transplant Research (CIBMTR) has addressed this aspect by comparing the cumulative incidence of CMV infection at day 180 in three cohorts: one that had received HaploCy (42%), a second group of sibling SCTs with PTCy (37%) and a third cohort of sibling SCTs with calcineurin inhibitor-based (23%) prophylaxis for AML/ALL/MDS. PTCy, regardless of donor, was associated with a higher incidence of CMV infection. The study also concluded that CMV infection could negate the cGVHD protective benefit of PTCy (75).

#### Methotrexate

Methotrexate is an antitumor and immunosuppressive drug. It is a structural analogue of folic acid; it blocks purine synthesis by inhibiting numerous regulatory enzymes. It does not have protumoral activity, unlike alkylating drugs, so it is of particular interest in the context of patients undergoing multiple therapies with potential induction of secondary tumours. It targets the S phase of the cell cycle, which determines that its action is largely confined to highly proliferative cells. In the early post-transplant period, it predominantly acts on highly proliferative alloreactive lymphocytes. Its use in the context of marrow transplantation dates back to 1970, when Donald Thomas described its role in controlling GVHD in dogs (76). Since then, its use has been maintained with dose modifications and optimizations in its combinations. In combination with a calcineurin inhibitor, it has been the standard of care for immunosuppression in myeloablative matched hematopoietic cell transplants.

#### Calcineurin Inhibitors

Calcineurin inhibitors stop downstream signalling of the T cell receptor (TCR) of naïve and memory T lymphocytes. This makes them highly effective at suppressing alloimmunity after SCT (77). They have undesirable collateral effects on anti-infectious and tumour-protective immunity, and reactivation of latent herpes viruses including CMV is frequent (78).

#### Anti-Thymocyte Globulin (ATG)

Low-dose ATG in transplants from high-risk alternative donors reduces GVHD and transplant-related death. All four randomized ATG trials undertaken demonstrated protection against GVHD, and three of them found no detrimental effect on survival (79–81). Two ATG formulations, derived from horse and rabbit, have different mechanisms of action, effects on Tregs, and depths of induced lymphopenia.

A direct association has consistently been found in both formulations between the use of ATG and the occurrence of viral infections, particularly CMV. This association has recently been validated (72).

##### Steroids

Patients being treated with high-dose steroids in the setting of GVHD, had significantly fewer activated CMV-specific T lymphocytes, both CD8+/IFN-γ+ and CD4+/IFN-γ+ at all developmental stages after allo-SCT. Reconstitution of CMV-specific CD4+ and CD8+ activated lymphocytes was observed at +180 days post-transplant, which was 80 days later than what was observed in the non-steroid counterpart (83). This work explains how and why steroid treatment increases the risk of CMV infection in patients who, because of their serological status and graft source, would otherwise not be at high risk of reactivation.

Some other groups have analysed how GVHD treatment affects T lymphocyte functionality. Young patients with active GVHD (based mainly on steroids) do not have adequate levels of activated CMV-specific CD4+and CD8+T lymphocytes, and do not produce IFN-γ and IL-2 (51). The lack of control of CMV reactivation after allogeneic SCT has been shown to respond to an impaired function of antigen-specific CD8+ T lymphocytes, whereas the amount of CMV-specific T lymphocytes does not have such a marked impact (84).

### Monitoring CMV Infection

Serological determination of CMV-specific antibodies is important for determining a patient’s risk of CMV infection after transplantation (85). However, it is worth noting that in polytransfused patients (as exemplified by many haematological patients awaiting an allogeneic transplant), the serological status may be an artefact of a passive immunization mechanism. Discrepancies in CMV have been observed in up to 29%-33% of patients when this has been analysed (86, 87). Routinely determined serostatus is still currently used as a criterion for estimating CMV reactivation risk before transplant.

Without prophylaxis, the disease of 80% of patients who are serologically positive for CMV would reactivate after allogeneic transplantation. Strategies to prevent the development of CMV disease have emerged in recent decades, based on antiviral prophylaxis and CMV viremia monitoring (before developing the disease), and treatment before the disease causes organ damage (pre-emptive therapy). Serological status is not appropriate for the purpose of detecting CMV infection. In turn, detection of CMV in blood is the recommended strategy for preventing CMV disease (88). It can be detected with pp65 antigen in leukocytes, or by the polymerase chain reaction (PCR) technique, which is more sensitive (89) and therefore the most frequently used. The presence of antigen in peripheral blood or high loads of DNA are both predictive of CMV disease in these patients (90, 91). Likewise, patients who are to receive SCT must receive leukodepleted, filtered and irradiated products.

### Treatment Options for CMV Infection: Improving Anti-CMV Reconstitution

To decide the best therapy for each patient, we must consider whether the patient has received antiviral prophylaxis, the risk profile for CMV disease, T lymphocyte reconstitution (both general and CMV-specific), viral load and potential drug resistance (92).

Effective agents to control CMV infection have been notably toxic. The three main drugs used in recent years are ganciclovir/valganciclovir, foscarnet and cidofovir. Ganciclovir is an analogue of nucleoside 2’-deoxyguanosine, which functions as a competitive inhibitor with deoxyguanosine triphosphate (dGTP) used by DNA polymerase of viruses for its replication. Foscarnet reversibly blocks the pyrophosphate-binding site of viral polymerase in a non-competitive manner and inhibits the separation of pyrophosphate from deoxynucleotide triphosphates, 100 times more strongly than its action against cellular DNA polymerase α. Cidofovir, in its active form of cidofovir diphosphate, prevents viral replication by selectively inhibiting viral DNA polymerases. It also inhibits human DNA polymerases, but up to 600 times weaklier. Ganciclovir induces haematological toxicity, in which neutropenia contributes to the development of opportunistic bacterial and fungal infections (93, 94). Foscarnet and cidofovir cause kidney damage and require strict analytical control that usually requires the patient’s admission to hospital to receive the treatment (95, 96). They are often used in the context of pre-emptive therapy, and, in fact, when oral agents such as valganciclovir are used as primary prophylaxis, there is no significant benefit (97). The strategy used to date has therefore been pre-emptive therapy, except in patients with a high risk of CMV disease, for whom alternative strategies are warranted. This scenario might soon change with the introduction of letermovir, which is known to reduce CMV reactivation and decrease all-cause mortality, but without being significantly toxic. In fact, it has performed similarly to the placebo in the phase 3 trials (98).

Letermovir inhibits the CMV DNA terminase complex, which is required for cleavage and encapsidation of the resulting viral DNA, thereby interfering with virion formation and without significant toxicities compared to placebo (98). This is extremely important since both ganciclovir, cidofovir and foscarnet have well known hematologic and renal toxicities that frequently limitate their use. The rationale for its potential benefit in comparison with other drugs is a different therapeutic target, that could overcome the resistance observed in the clinic (92, 99). There is an urgent need of drugs that effectively treat CMV reactivation, both in patients who are refractory to ganciclovir and in those who do not admit additional toxicities induced by the antivirals used. For this reason its use has been approved by some regulatory agencies and in the coming years we will verify the real impact on the clinic of transplant patients, fundamentally.

To improve T lymphocyte recovery and CMV control, strategies that aim to improve thymus function could be key. These include protection of thymic epithelium, thymopoiesis enhancement and increasing the number of T lymphoid precursors (47). These *in vivo* strategies have been highly diverse, including the use of specific lymphokines, growth factors and hormones, among others. On the other hand, cellular therapies have also been developed. Among these, infusion of *ex vivo*-expanded virus-specific cytotoxic T lymphocytes (CMV-VSTs) has been of particular note (100). These specific cytotoxic lymphocytes have been used either with the original donor source or with partially matched donors. In general, this strategy has proved efficacious in post-transplant CMV reactivation and disease (101, 102). There appear to be correlations (based on retrospective studies) between the baseline CD4+ level (the recipient’s previous immunity) and the rate and duration of engraftment and treatment success, probably because the CD4+ component is essential for mediating the engraftment and activity of the effector cells (103).

Attempts have been made to vaccinate against CMV, the most recent using techniques being based on DNA vaccines as well as peptide-based CMV conjugated with TLR agonists (104). The most advanced vaccines are those combined from CMV glycoprotein B (gB) with the adjuvant MF59. Its use has been tested in transplant patients to prevent post-transplant CMV disease, and in seronegative pregnant women or adolescents. In the first cohort (phase 2 study) of vaccinated *vs* placebo (105), a decrease in viremia was demonstrated in vaccinated patients proportional to the antibody load generated, although we know that the humoral response constitutes only one line of defence against the CMV, the results were not negligible, and confirmation of efficacy is awaited in phase 3 studies. In the cohort of young women, the vaccine was safe and immunogenic, although with an efficacy of 45% after 2 doses, therefore which was considered insufficient to continue the line of research (105). It is highly likely that the advances in DNA and RNA vaccine technology during 2020 and 2021 will change the spectrum of vaccines, and that the landscape will change in the years to come.

### CMV in Other Haematological Settings: CMV Reactivation and Drugs in Haematological Malignancies

#### CMV and Autologous Stem Cell Transplantation

The role of CMV reactivation or infection has been much less extensively studied in patients receiving an autologous transplant than in those receiving an allogeneic transplant. From the perspective of the treatment of haematological disease, which usually have high short-term mortality rates, CMV reactivations have been studied to determine whether or not the disease will develop. The implications of these reactivations for the immune system in the medium and long term have been considered less important. In fact, the available series confirm that reactivation is a relatively frequent event in patients receiving autologous parental transplants. CMV reactivates in up to 41% of patients during the post-transplant period. Anyway, the rate of CMV disease remains low (106). Those patients receiving high doses of steroids, irradiation, purine analogues or alemtuzumab would require more attention (107).

#### CMV and Lymphoproliferative Disorders

The immunosuppression observed in many haematological tumours is conditioned by the underlying disease itself, but also fundamentally by the type of treatment used. Lymphoid pathology (acute and chronic) reveals a fundamental involvement of the B and T lymphoid compartments. This creates a tendency to develop viral and fungal infections, as well as infections borne by some intracellular parasites, such as *Pneumocystis jirovecii*. The cellular immunosuppression observed in this group of patients determines many of the prophylaxis strategies, which are sometimes conditioned to the treatment, but in others are more typical of the immune defect that generates the disease.

Thus, trimethoprim/sulfamethoxazole prophylaxis and the use of acyclovir in lymphoproliferative syndromes are quite widespread.

Acute and chronic myeloid pathologies, such as acute leukaemias or myelodysplastic syndromes, involve the myeloid compartment. Neutropenia mainly conditions bacterial and fungal infections. Their long evolution, the use of purine analogues in treatment regimens, or the frequent consolidation with allogeneic transplantation in this group causes CMV infection to be a concern in more advanced stages of the disease. CMV serostatus and non-relapse mortality rate after transplant are quite well established in acute myeloid leukaemias (108). In turn, recent explorations of series featuring other pathologies such as diffuse large B-cell lymphoma (DLBCL) have not been able to demonstrate such an association (109).

B-cell chronic lymphocytic leukaemias (B-CLL) is the most frequent chronic leukaemia in western countries. It has been suggested that this disease features a CD8+ T lymphocyte expansion that increases as the disease advances. Analysis of specific immune responses with tetrameric CMV-peptide complexes showed that patients exhibiting such an expansion, actually have an increase of CMV-specific CD8+CD45RA+CD27- T lymphocytes. This change is actually specific to CMV-seropositive patients and might reflect the greater effort required to control CMV reactivations (110). Similarly, another analysis of CMV in CLL patients has revealed that the expanded CMV-specific response observed in CLL patients apparently arises with the onset of chemotherapy and stabilizes thereafter (111). Some researchers have called attention to the possible consequence of redirecting autologous CMV-specific cytotoxic T lymphocytes (CTLs) towards B-CLL cells for cancer immunotherapy (112). CMV infection in patients with CLL usually occurs in the context of treatment with purine inhibitors, alemtuzumab, or even alkylating agents such as chlorambucil, and the disease itself. Of all the mechanisms involved in CLL immunosuppression, which falls beyond the scope of this review, hypogammaglobulinemia seems to be of great importance. It has been thoroughly described in CLL and has recently been associated with a shorter time until the next treatment (113).

#### CMV and Purine Analogues

All purine analogues mimic metabolic purines. Of them, fludarabine is the most extensively used in hematologic cancer. Fludarabine inhibits multiple DNA polymerases, DNA primase, and DNA ligase I, and is S phase-specific (since these enzymes are highly active during DNA replication). Fludarabine acts particularly on T cells, and is therefore very toxic to this compartment.

Normal T lymphocyte function is essential to the control of CMV reinfection. The use of agents such as purine analogues, that have a very potent T lymphocyte suppressor profile while being highly effective for treating chronic lymphoproliferative disorders (114), are highly relevant to the development of CMV reactivations.

Cases of CMV retinitis have been documented in patients with low-grade lymphomas treated with rituximab, fludarabine and steroids (115). Previous serological status was not available for most patients, probably because there is not a specific approach with CMV seropositivity outside transplant scenarios. Despite being a very rare event even in the seropositive population, the sequelae were adverse (blindness) in 86% of patients. CMV monitoring, and clinical observation of possible infection and reactivation, must be considered when these regimens are used.

#### CMV and Alemtuzumab

Alemtuzumab is a monoclonal antibody which acts on the CD52 protein found on the surface of peripheral blood and lymph node lymphocytes.

CMV reactivations in patients receiving alemtuzumab treatment for CLL are common, with a rate of around 20% in the phase II study with the drug that was evaluated in 78 patients (116). However, viral load monitoring and pre-emptive treatment manages to prevent the disease in most cases (117).

Some studies have even proposed the use of primary prophylaxis with valganciclovir in patients receiving alemtuzumab in whom the drug has a high efficacy (118). However, the main concern with using prophylactic valganciclovir is the additional myelosuppression beyond the already significant amount produced by the alemtuzumab-based regimens themselves. An attempt to reduce cost and toxicity by using lower weekly doses (119) produced lower toxicity and acceptable efficacy. However, due to the aforementioned concerns, primary prophylaxis is not common practice in this group of patients.

#### CMV and New Agents in the Treatment of Lymphoproliferative Syndromes

Many drugs have been introduced into the therapeutic arsenal to treat lymphoproliferative syndromes, in particular, CLL. However, CMV seropositivity in the era of new therapies has not led to reduced survival or relapse in CLL patients when prospectively compared with seronegative patients (120).

### Bruton Tyrosine Kinase Inhibitors

CMV infection is not common in the case of a BTK inhibitor such as ibrutinib, although some cases have been reported (121). Although these are infrequent, we should always be aware of this possibility among immunocompromised patients, particularly those who have been treated with new agents, because this is a curable condition and early therapy seems to be critical if a good outcome is to be achieved.

### PI3K Inhibitors

Recommendations for the management and prophylaxis of CMV reactivation have been established in patients treated with idelalisib. These patients can develop a serious infectious disease, with a high risk of CMV reactivation and the involvement of other opportunistic germs (122). It has been shown how idelalisib impairs IFN-γ production by activating T lymphocytes in CLL-treated patients, highlighting the importance of PI3Kδ in this process. Idelalisib inhibits T lymphocytes in relation to generic TCR stimulation and in response to virus-specific stimulation. The CD4+ and CD8+ T lymphocyte subsets both seem to be affected. This might explain the higher rate of CMV reactivations in those CLL patients who are treated with it. Finally, idelalisib has also been shown to alter T lymphocyte migration *in vitro* (123).

The high risk of CMV reactivation in this therapy group has meant that the guidelines have been adapted to those for the use of alemtuzumab (124). CMV-seronegative patients should receive CMV-negative or filtered blood products (this is blood, platelet, and plasma transfusions. Progenitor cell transplantation products are not irradiated due to the need to maintain the viability of the infused cells). Seropositive patients should be periodically tested for CMV. Idelalisib should be discontinued and ganciclovir or valganciclovir pre-emptively initiated in patients with positive CMV PCR/antigen and symptoms consistent with CMV infection, as well as in patients with fever and no clear source, and for whom quantitative CMV testing is unavailable, and in asymptomatic patients with a rising viral load (125).

### Proteasome Inhibitors

Bortezomib and the next-generation proteasome inhibitors have been cornerstones of multiple myeloma (MM) treatment for several years. Although sufficiently relevant to be considered one of the causes of the increase in survival in this group of patients, they have also entailed infectious risks that should be highlighted. Bortezomib was reported to increase the risk of varicella-zoster virus (VZV) reactivation by up to four times (126), and a high incidence of CMV reactivation in fit patients receiving autologous transplantation due MM after treatment with bortezomib-based regimens was recently reported. Of 80 patients who underwent autologous SCT after bortezomib-based therapies, seven (4.1%) received an antiviral treatment for a symptomatic CMV reactivation and one died (127). Although no specific cause has yet been determined, *in vitro* studies have demonstrated that bortezomib can reduce proliferation, number, and function of natural killer cells and CD8+/CD4+ T and alter the cytokine profile, in particular decreasing Th1 cytokine secretion (128).

In the setting of allogeneic transplantation, bortezomib induces preferential apoptosis among alloreactive T lymphocytes (decreasing Th1 response) by inhibiting nuclear factor-κB (NF-κB) activation, whilst unstimulated T lymphocytes are barely affected. This might explain its potential use as a drug for GVHD treatment.

All these mechanisms could help explain the increased incidence of Herpesviridae family viruses (of which VZV is the best known) in MM patients and might contribute to the increased susceptibility to CMV reactivation in MM patients treated with bortezomib-based regimens followed by ASCT (129).

#### CMV and CAR-T-Cells

One of the most significant innovations in recent years has been the treatment with the CD19-directed chimerical antigen receptor T lymphocyte. Currently, data regarding the rate and type of viral infections in patients receiving this treatment are scarce. However, there are already some data on the incidence of CMV infections and reactivation in real clinical practice. In a retrospective series of 60 patients with aggressive lymphoma treated with CAR-T, who received antiviral prophylaxis for herpes simplex virus (HSV) according to the recommendations of each hospital, 10 viral infections were documented in the first 30 days, of which two corresponded to CMV reactivations, without organ affectation. During days 31-180, another reactivation by CMV occurred, again without organ disease. None of them, therefore, compromised the life of the patient (130).

Considering now the patients with ALL, data were collected from 83 patients up to 21 years of age who had received CAR-T therapy for this reason (131). Sixteen of them developed viral infections within the first 28 days; these were caused mainly by respiratory viruses, without specifying infection by CMV in these reported cases. Between days 29 and 90, seven patients got viral infections, all of which were due to respiratory viruses.

There are documented cases of CMV reactivation in the first month and during the first three months. Previous therapies, disease stage and patient basal characteristics seem to be crucial.

Regarding prophylaxis against viral infections, there are no unique international recommendations, and these recommendations are heterogeneous (132). The European recommendations are based on data from allogeneic transplant recipients (133). In general, antiviral prophylaxis is established with acyclovir or valacyclovir at least up to one year after CAR-T infusion, or until a CD4+ T lymphocyte level greater than 0.2 x 10^9^/L is documented (134). In paediatrics, nonspecific immunoglobulins are also frequently used to maintain total IgG levels above 0.4 g/L (135). With respect to monitoring, the EBMT guidelines recommend that PCR be performed when clinically indicated (133).

## Conclusions

The relationships between CMV infection and haematological pathologies are well known. Fundamentally, as a result of the important repercussions from the management of the infection and reactivation of the virus in the post-allogeneic transplant. However, there are many other situations that give rise to severe immunosuppression, either due to the haematological pathology itself or to the treatments used, which should increase vigilance concerning the complications derived from infection by this virus. Thus, it is necessary to study the effect of new drugs on the immune system and so adapt CMV prophylaxis and infection monitoring to different treatment schemes and situations, now that new anti-CMV drugs with fewer secondary effects are available for this purpose.

In contrast, knowledge about the role of this virus in the development of haematological diseases, in other words, its oncogenic or oncomodulatory potential, is much more limited. The difficulty in finding it may be because CMV infection is associated with age and the fact that its main effect is to bring about the dysfunction of T lymphocytes. T cell lymphomas are quite rare. The search for causality in the more commonly occurring counterpart B-cell lymphomas involves looking for indirect causes arising from the basic relationship between T and B lymphocytes.

Living with this herpesvirus is a situation that has arisen from years of evolution, which has apparently produced a balanced and tolerable relationship. This would imply that its effect, does not directly or indirectly limit survival or favour oncogenesis. In the forthcoming era of medicine we are approaching, there will be infinite possibilities for producing antiviral agents with very low toxicity and for immunizing against the most prevalent microorganisms. These promising possibilities should inspire an exhaustive study of the real effects of CMV and other microorganisms on the oncogenesis and mortality of individuals and populations. It seems clear that the ultimate answer about the oncogenic role of the virus will come from a prospective design approach that will allow us to determine whether human beings who are not infected have a lower risk of developing haematological neoplasms or other conditions than those who are infected.

## Author Contributions

The authors’ contributions were as follows: SA-Á and EC designed the study, SA-Á and EC wrote the manuscript, and MM-G and RA-A reviewed the manuscript. All authors contributed to the article and approved the submitted version.

## Funding

This work was supported by grant PI17/00714 from the Spanish I+D+i 2013–2016 State Program, which was jointly funded by the Instituto de Salud Carlos III and the European Regional Development Fund (ERDF).

## Conflict of Interest

The authors declare that the research was conducted in the absence of any commercial or financial relationships that could be construed as a potential conflict of interest.

## Publisher’s Note

All claims expressed in this article are solely those of the authors and do not necessarily represent those of their affiliated organizations, or those of the publisher, the editors and the reviewers. Any product that may be evaluated in this article, or claim that may be made by its manufacturer, is not guaranteed or endorsed by the publisher.
